# A-Lines below the Diaphragm – A Case Report of Occult Pneumoperitoneum

**DOI:** 10.5070/M5.52389

**Published:** 2026-07-31

**Authors:** Yong Hoon Lee, Michael Dalley

**Affiliations:** * Mount Sinai Medical Center, Department of Emergency Medicine, Miami Beach, FL

## Abstract

This case report highlights the rapid and sensitive diagnostic capabilities of a right upper quadrant (RUQ) ultrasound to diagnose pneumoperitoneum secondary to a hollow viscus rupture. The patient was a 55-year-old male with sudden onset epigastric and right upper quadrant pain. Symptoms were concerning for either acute cholecystitis and gastric ulcer or perforation. Immediate point of care ultrasound (POCUS) of the RUQ showed no signs of acute cholecystitis but showed concerning findings of intraabdominal free air given the enhanced peritoneal stripe sign, reverberation, and ring down artifacts. The radiologist did not note any signs of free air on the interpretation of the formal ultrasound. Eventually, the chest X-ray and computed tomography (CT) of the abdomen and pelvis showed air under the right diaphragm, fluid in the porta hepatis, and multiple foci of pneumoperitoneum. Broad spectrum antibiotics and fluid resuscitation were given. General surgery performed an emergency diagnostic laparotomy with repair of duodenal ulcer perforation. Upper gastrointestinal (GI) study on post-operative day three showed no complications, and patient has been doing well in follow-up clinic visits. In this case report, immediate recognition of signs of pneumoperitoneum would have expedited the treatment and management of the duodenal perforation. Although our emergency department was able to perform a CT scan of the abdomen and pelvis and have it read within two hours, this may not be possible in all emergency departments. Therefore, the use of POCUS in the evaluation of abdominal pain can identify potentially disastrous time-dependent pathology. It would be beneficial for emergency physicians to learn the signs of pneumoperitoneum on ultrasound since this requires emergent surgery.

**Topics:**

Hollow viscus perforation, ultrasound, pneumoperitoneum, point-of-care ultrasound (POCUS).

**Figure:**
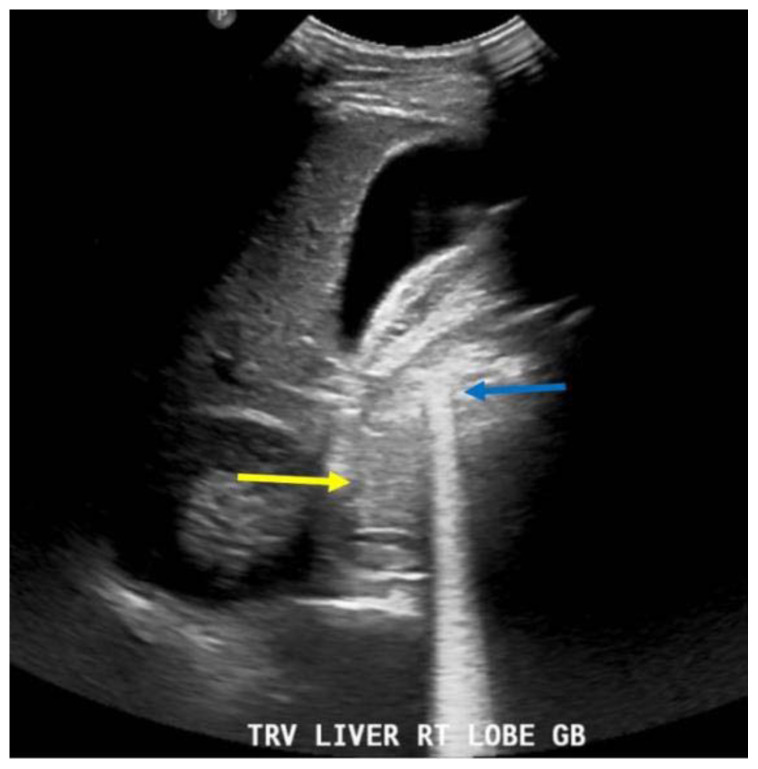


**Figure:**
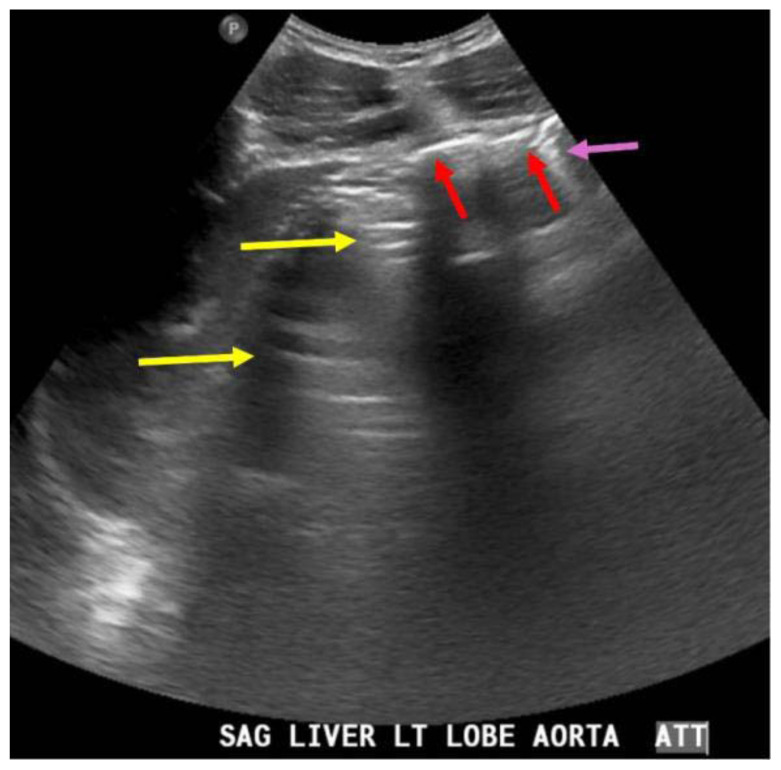


**Figure:**
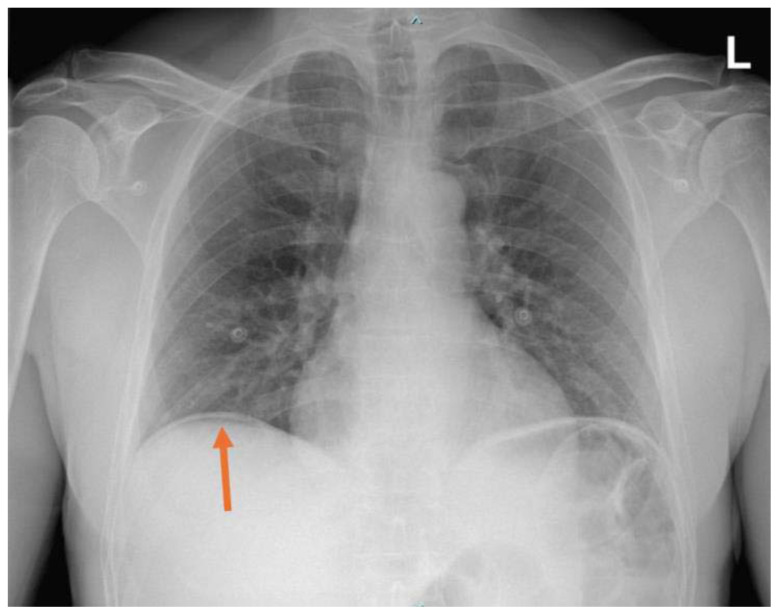


**Figure:**
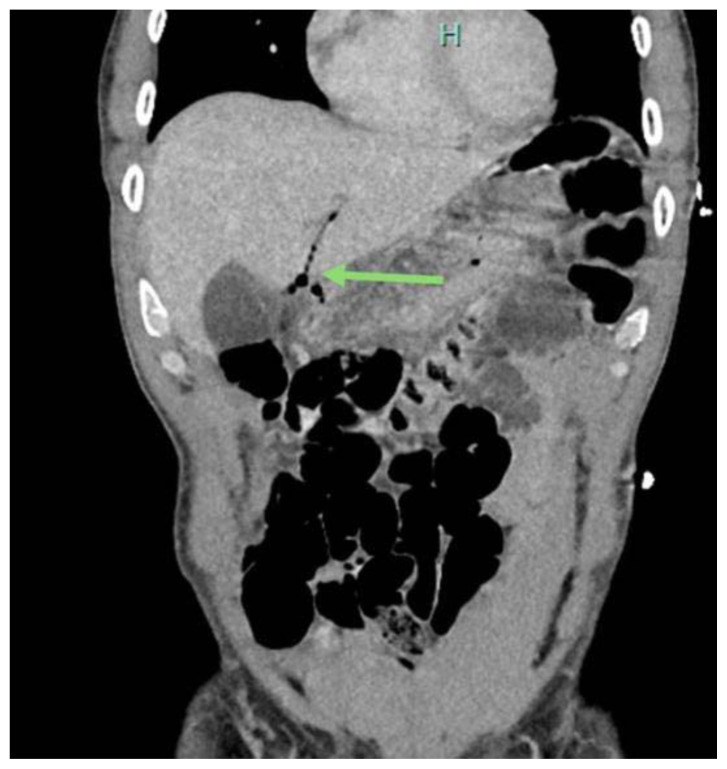


## Brief introduction

Hollow viscus perforation, specifically duodenal perforation, is a rare condition but has a high mortality rate.^1^,^2^ Peptic ulcer disease (PUD) is the most common cause of duodenal perforation and non-steroidal anti-inflammatory (NSAID) drug use is one of the most common causes of PUD.^3^ Around two percent of the population are affected by PUD with five percent of these patients experiencing a perforation in their lifetime.^4^,^5^ Symptoms of perforation typically include a sudden onset of abdominal pain, tachycardia, and abdominal rigidity.^6^ Upright chest radiographs (CXR) can identify a perforation with the presence of pneumoperitoneum, free air in the abdomen, by identifying air below the diaphragm in 75% of patients.^7^ The “gold-standard” for detecting intraperitoneal free air is the abdominal computed tomography scan with a sensitivity of about 81–100%.^8^ Delays in treatment have been shown to increase mortality, and around 30% of patients with perforations have sepsis on arrival to the operating room.^9^,^10^

Point-of-care ultrasound (POCUS) is an essential tool in the armamentarium of an emergency physician and can be used to quickly and accurately evaluate a patient with sudden onset abdominal pain. The differential is broad in many cases, ranging from abdominal aorta aneurysm, dissection, or rupture to cholecystitis. Using POCUS is an efficient way of narrowing the diagnosis immediately at the bedside without waiting for a portable radiograph or transporting an unstable patient to the CT. Therefore, identification of signs of pneumoperitoneum, including ring-down artifacts, enhancement of the peritoneal stripe, and reverberations, is an essential component of the abdominal ultrasound exam.^11^ With adequate training, ultrasound imaging has been found to be superior to radiographs in diagnosing pneumoperitoneum, with a sensitivity of 93%.^11^

## Presenting concerns and clinical findings

The patient is a 55-year-old male with no past medical history who was brought to the Emergency Department (ED) by Emergency Medical Services for sudden and severe RUQ abdominal pain. The pain started 30 minutes prior to arrival with a sudden onset of severe pain while he was talking to his daughter. Pain was described as pleuritic and intermittent. Associated symptoms include waves of nausea that worsen with his pain but no vomiting. Pain did not radiate to the rest of the abdomen, chest, or back. He did not take anything for the pain at this time.

He had been taking ibuprofen for a couple of months for a recent oral surgery. He also reported mild abdominal pain and non-bloody diarrhea for the past five days that he attributes to antibiotics from the surgery. Denied headache, flank pain, trauma to the abdomen, and history of gastric ulcers.

In the emergency department, he was afebrile, heart rate was regular at 92 beats per minute, oxygen saturation was 98% on room air, blood pressure was 132/91 mmHg. On exam, he appeared uncomfortable and clutching the right upper quadrant of his abdomen. His abdomen was guarded and mildly tender around his epigastric region and RUQ with positive Murphy’s sign. The differential was broad to include any pathology that can cause sudden RUQ pain such as pneumothorax, gastritis, cholecystitis or biliary colic, pancreatitis, and aortic dissection and hollow viscus perforation. Laboratory diagnostics including complete blood count, basic metabolic panel, hepatic function panel and lipase levels were within normal limits. A CXR and formal RUQ ultrasound were ordered, but a POCUS of the RUQ was done immediately at bedside to assess for cholecystitis to prevent delay of care. After the POCUS, the comprehensive RUQ ultrasound and CT of the abdomen and pelvis with intravenous contrast was done.

## Significant findings

Point-of-care right upper quadrant ultrasound was performed by the ED physician (Mindray C5-2s 2–5 MHz Curved Array Probe) and the gallbladder was unremarkable, with no wall thickening, cholelithiasis, pericholecystic fluid.

The RUQ POCUS focusing over the left liver lobe shows multiple enhanced peritoneal stripe signs (red arrows) and several reverberation artifacts (yellow arrows). The second RUQ POCUS of the right lobe of the liver and the gallbladder shows reverberation artifacts (yellow arrow) and ring-down artifacts (blue arrow). The chest X-ray shows free air under the right diaphragm (orange arrow), which indicates pneumoperitoneum. The CT abdomen and pelvis show pneumoperitoneum near the porta hepatis (green arrow).

## Patient course

Given the sudden RUQ pain, the differential was focused more on cholecystitis than a perforated ulcer, particularly since the patient denied significant abdominal pain prior to today. All labs were otherwise within normal limits. We noted the abnormal findings on the ultrasound, but due to our inexperience and thus lack of confidence diagnosing pneumoperitoneum on ultrasound, we delayed our diagnosis to consult surgery until the CXR and CT confirmed pneumoperitoneum. In our community hospital, it took an hour for the patient to get the CT and an hour longer to be read by the radiologist. Broad spectrum antibiotics, piperacillin-tazobactam (4.5 g loading dose IV), and fluids were given. General surgery then performed an emergency diagnostic laparotomy with repair of a duodenal ulcer perforation. Upper GI study on post-operative day three showed no leak, and patient was discharged home without complications. At the most recent post-operative clinic visit, patient states no fever or pain and tolerates oral intake without complication.

## Discussion

Pneumoperitoneum due to a duodenal ulcer perforation is an uncommon finding amongst patients with epigastric or abdominal pain (less than one percent) but should be discovered early due to increased morbidity and mortality from delays (30-day mortality rate of 20–50%).^10^,^12^ Although gastrointestinal perforations are rare, patients with peptic ulcer disease, frequent NSAID use, or diverticulitis have the most common causes of perforations (16% each).^13^ When considering POCUS to evaluate for pneumoperitoneum, studies have shown that it has improved sensitivity over radiography (86–94% vs 78%) and variable specificities (53–64% vs 53%).^14^,^15^ However, operator proficiency is important for accurate diagnosis; a study by Nazerian et al. showed that, when going through a short training course to recognize pneumoperitoneum, physicians experienced in ultrasound had a diagnostic accuracy of 89% compared to 68% of physicians who were not familiar with ultrasound.^16^ Although CT of the abdomen is the gold standard with a sensitivity of 81–100% and a specificity of 97–100%, long delays to scan, radiation exposure, and high costs can make it a less desirable modality.^8^

Intraperitoneal free air is best detected in the RUQ between the anterior abdominal wall and the liver where there are no intervening bowels. In terms of identifying signs of pneumoperitoneum, there are three main findings to take note of. If there is air within the peritoneal space, an enhanced peritoneal stripe sign (EPSS) can be seen (image 1). Normally, the transition of ultrasound waves through the anterior abdominal wall into the peritoneal fluid will highlight a thin peritoneal stripe that is echogenic to surrounding tissues. With free air between the peritoneum and the viscera, ultrasound waves are interrupted after passing through the anterior abdominal wall, causing scattering of the waves, and resulting in high-amplitude linear echoes that enhance the normal peritoneal stripe sign.^17^ Next, reverberation artifacts can be visualized deep to the peritoneal stripe. These are similar in concept and visually to A-lines in lung ultrasound exams. These are repeated reflections caused by an initial reflection at a highly reflective interface in the setting of free air, such as the abdominal wall and the peritoneum or the gallbladder wall and bowel (image 2).^18^ Comet tail artifacts are conceptually similar to reverberation artifacts but appear as a bright tapering trail of echoes from a strongly reflective structure (image 1).^18^ Ring down artifacts can also be seen as a long bright streak of echoes caused by a resonance from fluid trapped within a tetrahedron of air bubbles (image 2).^19^ It is difficult to discern between the comet tail and ring down artifacts, as both can show either a hyperechogenic line or a series of bands, but the physiology behind the presence of the artifacts is different.

Intraluminal bowel gas is a common false positive finding for free air. It is important to assess for peristalsis of the air as a marker for intraluminal bowel gas. Moving the patient from supine to left lateral decubitus will see movement of the EPSS and reverberation artifacts, whereas intraluminal air will not move from the confines of the bowel.^20^ The “scissor maneuver” or “curtain maneuver” is another technique to ensure whether the image artifacts are from free air: slight pressure with the probe expels the free air to other parts of the peritoneal cavity and improves visualization of the viscera. Releasing the pressure while maintaining contact of the probe to skin will return the free air to the RUQ and the artifacts return.^21^

This case is interesting and relevant to the practice of Emergency Physicians (EPs) because epigastric or RUQ abdominal pain is a common complaint in the ED (8.8% of all visits).^22^ Evaluation of epigastric or RUQ pain often involves working up cholecystitis or biliary colic, indicating a RUQ ultrasound. Many EPs are comfortable with POCUS RUQ ultrasounds and can quickly assess a patient when they initially evaluate the patient. Specifically, physicians proficient in ultrasound can receive a very short training course to recognize pneumoperitoneum with a “2 scan-fast exam” in the RUQ as demonstrated by studies from Chen et al. and Nazerian et al.^15^,^16^ This simpler exam utilizes a 2–5 MHz Curved Array probe that only scans the epigastrium and right hypochondrium with a patient lying supine and leaning onto their left flank. It was found to have similar sensitivity to a full RUQ exam, 87% vs 89%.^16^ Rapid diagnosis in this case would have led to quicker time to the operating room and reduce the rate of complications from delayed treatment. In our community hospital, treatment was delayed by two hours but in other hospitals, the delay could be several hours or even days longer with limited resources. With adequate training and an open differential, pneumoperitoneum can be quickly recognized within minutes to expedite care.

## Supplementary Information
















